# Pathological rate matrices: from primates to pathogens

**DOI:** 10.1186/1471-2105-9-550

**Published:** 2008-12-19

**Authors:** Harold W Schranz, Von Bing Yap, Simon Easteal, Rob Knight, Gavin A Huttley

**Affiliations:** 1 John Curtin School of Medical Research, The Australian National University, Canberra, ACT 0200, Australia; 2 Department of Statistics and Applied Probability National University of Singapore, Kent Ridge, Singapore; 3 Department of Chemistry & Biochemistry, University of Colorado, Boulder, CO, USA

## Abstract

**Background:**

Continuous-time Markov models allow flexible, parametrically succinct descriptions of sequence divergence. Non-reversible forms of these models are more biologically realistic but are challenging to develop. The instantaneous rate matrices defined for these models are typically transformed into substitution probability matrices using a matrix exponentiation algorithm that employs eigendecomposition, but this algorithm has characteristic vulnerabilities that lead to significant errors when a rate matrix possesses certain 'pathological' properties. Here we tested whether pathological rate matrices exist in nature, and consider the suitability of different algorithms to their computation.

**Results:**

We used concatenated protein coding gene alignments from microbial genomes, primate genomes and independent intron alignments from primate genomes. The Taylor series expansion and eigendecomposition matrix exponentiation algorithms were compared to the less widely employed, but more robust, Padé with scaling and squaring algorithm for nucleotide, dinucleotide, codon and trinucleotide rate matrices. Pathological dinucleotide and trinucleotide matrices were evident in the microbial data set, affecting the eigendecomposition and Taylor algorithms respectively. Even using a conservative estimate of matrix error (occurrence of an invalid probability), both Taylor and eigendecomposition algorithms exhibited substantial error rates: ~100% of all exonic trinucleotide matrices were pathological to the Taylor algorithm while ~10% of codon positions 1 and 2 dinucleotide matrices and intronic trinucleotide matrices, and ~30% of codon matrices were pathological to eigendecomposition. The majority of Taylor algorithm errors derived from occurrence of multiple unobserved states. A small number of negative probabilities were detected from the Padé algorithm on trinucleotide matrices that were attributable to machine precision. Although the Padé algorithm does not facilitate caching of intermediate results, it was up to 3× faster than eigendecomposition on the same matrices.

**Conclusion:**

Development of robust software for computing non-reversible dinucleotide, codon and higher evolutionary models requires implementation of the Padé with scaling and squaring algorithm.

## Background

The dynamics of genetic divergence are typically modelled as a Markov process where the rates of exchange between discrete sequence states are described by rate matrices. Discrete- or continuous-time Markov processes employ different, but related, rate matrices. The former involve a substitution matrix that specifies the probabilities of substitution between sequence states in a discrete period of time (**P**(*t*), [1]). The continuous-time Markov process employs an instantaneous rate matrix (**Q**), which defines the instantaneous relative rates of interchange between sequence states from which the substitution probabilities for a specified time period are obtained by **P**(*t*) = exp(**Q***t*) where *t *represents time and exp is the matrix exponential. The most commonly employed rate matrices impose the restriction that evolutionary processes are time-reversible (e.g. [1]). The inaccuracy of this restriction is shown by the specificity of particular mutagens and repair enzymes [2], and by the apparent directionality of amelioration of horizontally transferred genes to the background genome composition [3].

Relaxing the assumption of time-reversibility requires consideration of non-reversible matrices. Assessments of time-reversibility, which have largely been restricted to nucleotide rate matrices, show that non-reversible models can provide better estimation of important evolutionary parameters including rates of evolution and, when employed with the maximum-likelihood phylogenetic inference framework, phylogenetic tree support [4]. The development of approaches to identifying both biologically accurate and parametrically succinct models is therefore of considerable interest. Non-reversible forms of codon substitution models would allow, for instance, consideration of temporal changes in mutation pressure on natural selection. However, limitations of matrix exponentiation algorithms have been cited as motivation for continued development of reversible models (for example, [5]). Exploration of statistically efficient (parametrically reduced) forms might be most readily achieved using continuous time processes, but the accuracy of these approaches hinges on the properties of exponentiation of matrices from real biological data.

The most obvious method for computing a matrix exponential is the generalisation of the Taylor series expansion of a scalar exponential [6]. Instead of a series of scalar terms, the matrix exponential is expressed as a Taylor series over terms involving matrix products. The series is truncated at a sufficiently large finite number of terms (*M*).

$$\begin{array}{c} P(t)=\exp(Qt)\\ =I+Q(t)+\frac{Q^{2}t^{2}}{2!}+\frac{Q^{3}t^{3}}{3!}+\ldots \\ \approx \sum_{i=0}^{M}\frac{Q^{i}t^{i}}{i!} \end{array}$$

Convergence of the series expansion depends on the magnitude of the matrix norm ||**Q**|| (a measure of the size of the elements in **Q**, see Methods equation 1) [7]. An important property of the Taylor series is a reduced rate of convergence for matrices with large ||**Q**|| such that achieving a required accuracy involves increasingly larger *M*. A further problem is that the impact of roundoff error increases with term order so the method becomes impractical and inaccurate for matrices with large ||**Q**||. Although some efficiency improvements can be made to reduce the number of matrix products required [7], the essential defect remains. Accordingly, the Taylor series expansion performs worst on potentially the most biologically relevant matrices and thus sets a lower bound on both accuracy and computational performance. We will subsequently refer to the Taylor series matrices exponentiation algorithm as exp_TAYL_.

Several other algorithms compute the matrix exponential, but differ substantially in their computational behaviour, performance, and vulnerability to so called pathological matrices. For the purposes of this paper, we define a pathological matrix as one that results in a substantial discrepancy between the 'true' value of **P **and the value computed as exp(**Q***t*) by a given algorithm. It is important to note that a matrix property responsible for a discrepancy affecting one particular algorithm may not necessarily affect another algorithm to the same extent.

Of the collection of algorithms, that proposed as most robust (described below) is seldom adopted in the field of molecular evolution: instead, the method of matrix exponentiation by eigendecomposition is most widely used by existing software packages but is far less robust [6]. This latter algorithm is based on a matrix decomposition approach involving similarity transformations of the form **Q **= **SBS**^-1 ^so that

exp(**Q**) = **S **exp(**B**)**S**^-1^

where the aim is to find an **S **for which exp(**B**) is easy to compute. In the case of eigendecomposition, if **Q **= **UDU**^-1 ^where **U **are the eigenvectors, **D **is a diagonal matrix containing the eigenvalues of **Q **and **P**(*t*) the matrix of substitution probabilities for time *t*, then

**P**(*t*) = **U **exp(**D***t*)**U**^-1^.

The eigendecomposition approach has an important practical advantage for molecular evolutionary applications – the spectral theorem allows the calculation of arbitrary many values of exp(**Q***t*) from a single decomposition [7]. For a phylogenetic model that assumes a single (global) **Q **across the entire tree, for instance, this property means the decomposition need be performed only once per model evaluation. The *O*(*n*^3^) complexity of the decomposition is thus outweighed by its suitability for caching intermediate results. Eigendecomposition works well for normal matrices (where a normal matrix is defined as one that commutes with its conjugate transpose [7]) but breaks down when **Q **does not have a complete set of linearly independent eigenvectors (an invertible **U **does not exist) or when **U **is close to singular, i.e. when the condition number of the matrix of eigenvectors cond(**U**) = cond_EV_(**Q**) is large. (We illustrate the analytical conditions under which the eigendecomposition approach can fail with an example in Additional file 1.) We will subsequently refer to the eigendecomposition matrix exponentiation algorithm as exp_EIG_.

The algorithm advocated by Moler and van Loan [6] computes the matrix exponential using the Padé approximation in combination with scaling and squaring [8]. Padé approximants converge if the matrix norm ||**Q**|| is not too large. Thus, the idea is to reduce the matrix norm by scaling the matrix and then use Padé approximation (a ratio of series) to compute the scaled matrix exponential and then the full matrix exponential by squaring operations. The scaling and squaring operation reduces the norm of the matrix ||**Q**|| to that of a matrix ||**Q***'*||

**P**(*t*) = [exp(**Q***t*/*m*)]^*m *^= [exp(**Q***'t*)]^*m*^

The Padé approximation is a ratio of series where, typically, the series (denoted *p *and *q*) are constrained to be equal as diagonal Padé approximants are preferred for numerical efficiency [6]. The Padé approximation with scaling and squaring is then

$$\begin{array}{c} P(t)=[\exp{\left(Q^{\prime }(t)\right]}^{m}\\ ={\left(\frac{\sum_{j=0}^{p}\frac{(p+q-j)!p!}{(p+q)!j!(p-j)!}{\left(Q^{\prime }t\right)}^{j}}{\sum_{j=0}^{q}\frac{(p+q-j)!q!}{(p+q)!j!(q-j)!}{\left(-Q^{\prime }t\right)}^{j}}\right)}^{m} \end{array}$$

The diagonal Padé method with scaling and squaring requires of the order of *O*((*q *+ *m *+ 1/3)*n*^3^) operations but is, in general, more efficient than the Taylor series. To compute single values of exp(**Q***t*) therefore also takes of the order of *O*(*n*^3^) operations, but does not have intermediate results that can be cached. Thus, each tree branch (and unique value of *t*) requires an independent exp(**Q***t*). The robust computational performance of Padé therefore comes at the cost of requiring an independent exp(**Q***t*) computation for each value of *t*. We will subsequently refer to the diagonal Padé with scaling and squaring matrix exponentiation algorithm as exp_PADÉ _.

Given the evidence that the algorithms commonly employed by molecular evolutionary software can significantly err in their computation of the exponential [6], a survey of whether matrices pathological to these algorithms exist in nature is essential for the development of biologically realistic models of sequence evolution that are computationally robust. Here we report the results of a survey for matrices pathological to the exp algorithms in both protein coding and non-protein coding sequences from lineages as diverse as microbes and primates.

## Results

**P **matrices were derived from species triads composed of two ingroups and an outgroup. Knowledge of the outgroup allows determination of the sequence states ancestral to the ingroup lineages, thereby enabling a simple counting procedure for generating the **P **matrices (described in the Methods). The outgroup further allows the resulting matrices to be non-reversible. For the current comparisons we arbitrarily set the time *t *to 1.

For each **P**, the corresponding **Q **was estimated using a constrained optimisation procedure. While the relationship between **P **and **Q **suggested using the matrix logarithm to estimate **Q**, doing so resulted in nearly all dinucleotide and higher matrices having negative off-diagonal elements. In preliminary analyses, we determined that this property arose from sampling error (results not shown). Importantly, because matrices with negative off-diagonals cannot be readily interpreted we developed a constrained optimisation procedure for estimation of **Q **from $\hat{P}$. This procedure, which we describe in more detail in the Methods, used a numerical optimisation algorithm to minimise the value of ||$\hat{P}$- exp($\hat{Q}$)|| subject to the constraint *q*_*ij*, *i *≠ *j *_≥ 0. By default, this procedure employed the exp_PADÉ _algorithm, resulting in a bias towards this algorithm which we address in detail later. We note here that as the results were not substantively different when we used the matrix logarithm for estimating **Q **and the constrained optimisation approach produces matrices more likely to be representative of naturally occurring rate matrices, we only report results from the latter procedure.

To measure the magnitude of a matrix we employ the Frobenius norm [6], the square root of the sum of the absolute squared elements of a matrix (see Methods equation 1). The matrix norm is also used as a measurement of the error, or discrepancy, between $\hat{P}$ and the results of exp_TAYL_, exp_EIG _and exp_PADÉ _algorithms and we denote these error statistics as ϵ_*TAYL*_, ϵ_*EIG *_and ϵ_*PADÉ *_(see Methods equations 2–4). The additional matrix property of eigenvector matrix condition number, the product of the spectral matrix norms of the eigenvector matrix and inverted eigenvector matrix of **Q **(Methods equation 5), is also used as it indicates the suitability for digital computation of a matrix for eigendecomposition [9-11]. Finally, because the elements of **P **are probabilities, we also defined a matrix as pathological to an algorithm if it contained an invalid probability value (< 0, > 1).

### Pathological matrices in microbes

Orthologous protein coding gene sequences were sampled from all species triples in KEGG where neighbours were 0–2% distant by 16 S rRNA and outgroups were 2–10% divergent. This procedure resulted in 136 valid species triples. Given the large number of genes, and that preliminary work indicated estimating a single trinucleotide **Q **required nearly 1 day, we concatenated all alignments from a triad and estimated separate **Q **matrices for each ingroup lineage from these concatenated alignments. As described in the Methods, aligned codon columns with non-nucleotide characters (such as indels or ambiguous bases) were deleted. Nucleotide and trinucleotide samples were obtained from the unmodified resulting alignments. Two distinct dinucleotide samples were considered. The patterns of mutation are typically distinct between the three different codon positions due to their differing influence of variation in the encoded amino acid sequence. We therefore consider dinucleotides sampled from both the first and second or second and third codon positions. We refer to these as the dinucleotide 1+2 or 2+3 matrices respectively. The minimum, median and maximum filtered, concatenated alignment lengths were ~31 Kbp, ~1.5 Mbp and ~3.7 Mbp respectively.

Measures of exponentiation error for nucleotide matrices were low across all algorithms (Table 1). There were no exponentiation failures by any algorithm for the nucleotide matrices. A small fraction (< 1%) of exp_EIG _failed, however, for the dinucleotide matrices derived from codon positions 1+2 or 2+3, with one significant failure resulting from a large number of zero elements that lead to a singular eigenvector matrix, and four failures where the maximum element size resulted in an invalid probability. Almost all $\left(\frac{255}{257}\right)$ the trinucleotide matrices were pathological to exp_TAYL_, but none of these matrices were pathological to either the exp_EIG _or exp_PADÉ _algorithms. The other descriptive statistics increased with the dimension of the matrices: matrix norm and eigenvector matrix condition numbers systematically increased from the nucleotide to trinucleotide matrices. The distinct dinucleotide positions were predominantly consistent with each other, with median matrix statistics and error values of similar order.

**Table 1:** Exponentiation of matrices from microbes.

**Data**	**Norm^1^**	**Cond^1^**	**ϵ_TAYL_^1^**	**ϵ_EIG_^1^**	**ϵ_PADÉ_^1^**	$E_{TAYL}^{2}$	$E_{EIG}^{2}$	**N^3^**
Nuc	(0.062, 0.59)	(1.4, 4.6)	(7.1e-06, 2e-05)	(7.1e-06, 2e-05)	(7.1e-06, 2e-05)	0	0	272
Dinuc 1+2	(0.082, 1.9)	(4.7, inf)	(0.00012, 0.0032)	(0.00012, inf)	(0.00012, 0.0032)	0	3	272
Dinuc 2+3	(0.19, 3.1)	(3, 1e+02)	(0.00015, 0.0079)	(0.00015, 0.0079)	(0.00015, 0.0079)	0	1	272
Trinuc	(3.2e+02, 4.4e+02)	(19, 3.5e+02)	(3.7e+84, 1.5e+136)	(0.22, 0.23)	(0.22, 0.23)	256	0	257

^1 ^– median and maximum values; ^2 ^– Number of **P **matrices for the indicated algorithm with an invalid probability; ^3 ^– total number of matrices; inf – an infinite difference, typically arising from an exponentiation error.

### Pathological matrices in primates

Intronic alignments were sampled from Ensembl release 46 for 3 primate lineages – human, chimpanzee and macaque – where the outgroup status of macaque relative to the great apes is well established [12]. Intronic sequences were sampled due to increased confidence in their orthology arising from the relationships between exonic sequences. Sequences unlikely to evolve by a point mutation process (such as simple repeat sequence) were masked and all alignment columns containing non-nucleotide characters were removed (see Methods). There were a total of 1079 alignments with minimum, median and maximum filtered alignment lengths of ~30 Kbp, ~59 Kbp and ~438 Kbp respectively.

**Figure 1:**
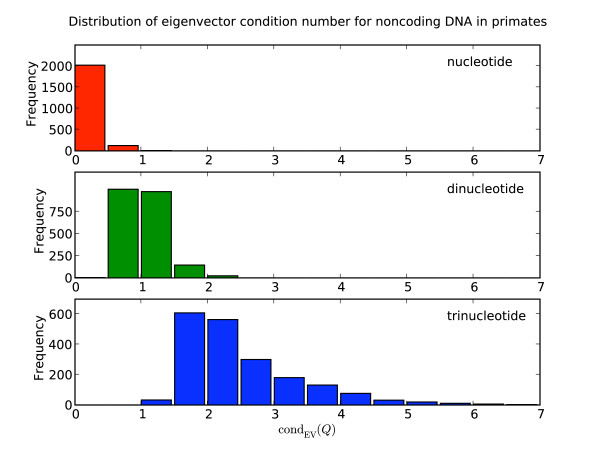
**Eigenvector matrix condition number increases with the dimension of the substitution model**. Data are from primate introns.

As for the microbial exonic sequences, no nucleotide matrices pathological to any of the algorithms were evident (Table 2). The results from dinucleotide matrices 1+2 and 2+3 were similar – low errors across all algorithms and no matrices pathological to any algorithm. This result is distinct from that evident from the microbial exonic data. Also different to the microbial data were an appreciable frequency (~10%) of trinucleotide matrices pathological to exp_EIG _while no matrices were pathological to exp_TAYL_. A moderate number of matrices were also pathological to exp_PADÉ_, but inspection of these revealed a maximum negative element size of -1.2e-33, indicating these as likely deriving from rounding errors. Also consistent with the results from the microbial analyses, the eigenvector matrix condition numbers increased from the nucleotide to trinucleotide matrices (see Figure 1).

**Table 2:** Exponentiation of matrices from primate intron sequences.

**Data**	**Norm^1^**	**Cond^1^**	**ϵ_TAYL_^1^**	**ϵ_EIG_^1^**	**ϵ_PADÉ_^1^**	$E_{TAYL}^{2}$	$E_{EIG}^{2}$	**N^3^**
Nuc	(0.015, 0.047)	(1.5, 56)	(6.8e-06, 1.7e-05)	(6.8e-06, 1.7e-05)	(6.8e-06, 1.7e-05)	0	0	2158
Dinuc 1+2	(0.074, 0.21)	(10, 3.3e+02)	(0.00016, 0.00058)	(0.00016, 0.00058)	(0.00016, 0.00058)	0	0	2158
Dinuc 2+3	(0.074, 0.2)	(11, 1.2e+03)	(0.00016, 0.00085)	(0.00016, 0.00085)	(0.00016, 0.00085)	0	0	2158
Trinuc	(0.24, 0.95)	(1.8e+02, 1.1e+07)	(0.001, 0.017)	(0.001, 7.5)	(0.001, 0.017)	0	188	2080

^1 ^– median and maximum values; ^2 ^– Number of **P **matrices for the indicated algorithm with an invalid probability; ^3 ^– total number of matrices.

Motivated by the differing frequency of pathological matrices between microbial exonic and primate intronic data, we sampled a collection of primate protein coding gene exons. Specifically, we used exonic sequence from a subset of the genes from which the introns were obtained. Given the restriction of pathological matrices to dinucleotide or higher alphabets, and high computational demands of fitting trinucleotide models, we sampled 1028 CDS alignments and combined these into 103 concatenated (10 loci each) alignments (see Methods). The minimum, median and maximum filtered, concatenated alignment lengths were ~12 Kbp, ~24 Kbp and ~40 Kbp respectively.

The matrix properties for these data, shown in Table 3, showed more consistency with those of the microbial exonic data than primate intronic data. As observed in the microbial analyses, an appreciable frequency (~8%) of dinucleotide matrices from codon positions 1+2 were pathological to exp_EIG _but not to exp_TAYL_. A smaller proportion (~1%) of matrices from codon positions 2+3 were pathological to exp_EIG_. Also consistent with the microbial exon analysis results, all trinucleotide matrices were pathological to exp_TAYL_. These results establish that the frequency of matrices pathological to exp_TAYL _and exp_EIG _depends on the class of sequence being sampled.

**Table 3:** Exponentiation of matrices from primate protein coding exons.

**Data**	**Norm^1^**	**Cond^1^**	**ϵ_TAYL_^1^**	**ϵ_EIG_^1^**	**ϵ_PADÉ_^1^**	$E_{TAYL}^{2}$	$E_{EIG}^{2}$	**N^3^**
Dinuc 1+2	(0.021, 0.16)	(42, 1.4e+04)	(3.1e-05, 0.00044)	(3.1e-05, 0.00044)	(3.1e-05, 0.00044)	0	17	206
Dinuc 2+3	(0.051, 0.18)	(23, 1.1e+04)	(7.1e-05, 0.00041)	(7.1e-05, 0.00041)	(7.1e-05, 0.00041)	0	3	206
Trinuc	(2.7e+02, 3.4e+02)	(2.9e+02, 2.3e+04)	(6.4e+65, 3.6e+89)	(0.22, 0.22)	(0.22, 0.22)	206	0	206
Codon	(0.15, 0.42)	(4.7e+02, inf)	(0.00049, 0.0029)	(0.0005, 0.087)	(0.00049, 0.0029)	0	62	206

^1 ^– median and maximum values; ^2 ^– Number of **P **matrices for the indicated algorithm with an invalid probability; ^3 ^– total number of matrices.

An important difference between the trinucleotide matrices from the intronic and exonic sequences is the presence of the trinucleotides encoding stop codons. We assessed whether inclusion of these states, which are absent from the in frame exons, contributed to the exponentiation errors by excluding unobserved states from the trinucleotide count matrices. This therefore generates 61 × 61 matrices, as employed by codon substitution models. As shown in the last row of Table 3, with the removal of unobserved states the errors from exp_TAYL _were completely eliminated. We further confirmed that the presence of unobserved states was responsible for the exp_TAYL _failures by taking a dinucleotide counts matrix and selecting two states to be missing (setting the corresponding row/column counts to all zeros). Exponentiation of this constructed matrix also proved pathological to exp_TAYL_. Interestingly, the frequency of errors from exp_EIG _was significantly increased with ~30% of codon matrices proving pathological (Table 3).

## Discussion

Our analyses confirm the numerical qualities of the three matrix exponentiation algorithms are distinct and that matrices pathological to both exp_EIG _and exp_TAYL _exist in nature. The magnitude of errors ranged from the subtle, **P **had probabilities close to but outside the interval 0–1, to extreme cases of algorithmic failure or extremely large elements. The range of these errors, and the data-type dependent frequency of matrices pathological to an algorithm indicate that exp_TAYL _and exp_EIG _are ill-suited to evaluation of non-reversible evolutionary models or to data where more than one sequence state is not observed.

An important impact of our study design is a bias towards exp_PADÉ _which we now partly redress. This bias was inevitable because estimates of **Q **had to be obtained from estimates of **P **and the prohibitive computational time required for estimation of trinucleotide **Q **necessitated a choice of a single exp algorithm. For the microbial lineages in particular, fitting a single trinucleotide **Q **frequently took ~1 day. We therefore elected to fit the trinucleotide matrices using the exp_PADÉ _since it was supported as most robust [6]. Nonetheless, we considered the bias introduced by estimating **Q **using one algorithm and computing exp(**Q**) with another. The dinucleotide model failures of exp_EIG _provided an opportunity to efficiently (with regards to compute time) assess whether constrained optimisation of **Q **using exp_EIG_, instead of exp_PADÉ_, might eliminate the matrices pathological to exp_EIG_. We therefore modified the constrained fitting of **Q **to use exp_EIG _instead of exp_PADÉ_, and applied the algorithm to the primate dinucleotide matrices derived from codon positions 1+2 of the concatenated protein coding sequences and exponentiated the resulting $\hat{Q}$ also using exp_EIG_. From the resulting matrices there was 1 failure during optimisation and 6 matrices remained pathological to exp_EIG _after this procedure. This number is smaller than the 17 failures (Table 3) for exp_EIG _when applied to **Q **estimated using exp_PADÉ _. These results indicate that although using a different algorithm for estimating **Q **introduces some bias, exp_EIG _still fails at an appreciable frequency. We note that for the **Q **matrices estimated using exp_EIG_, neither exp_PADÉ _nor exp_TAYL _exhibited high ϵ. However, the exp_PADÉ _of **Q **estimated by exp_EIG _returned invalid probabilities from $\frac{21}{4701}$ of the matrices examined in this study (all matrices considered in Tables 1, 2, 3 and those estimated using exp_EIG_). These failures were all extremely small negative values, the largest absolute value being ~10^-34^, likely reflecting rounding errors and thus their infrequent occurrence and extremely small size support the robustness of exp_PADÉ _.

The failure on both sequence classes of the eigendecomposition algorithm can originate from properties of the eigenvectors and/or eigenvalues. If the eigenvector matrix of a given **Q **is near-singular, then spectral expansion of the matrix exponential exp_EIG_(**Q**) is ill-defined. The eigenvalues influence on the computations occurs when a **Q **has very many almost degenerate (in the examples in the present study, near zero) elements. In these cases, though the numerically determined eigenvector matrix has an inverse, the set of numerically determined eigenvalues and eigenvectors do not accurately satisfy the eigenvalue equation [13] that can lead to failure of the spectral representation of the matrix exponential exp_EIG _of **Q**.

There appears to be a connection between these two types of failures: matrices with ill-conditioned eigenvector matrices lie close to ones with multiple eigenvalues [14]. Thus, the eigendecomposition can fail (or become inaccurate) due to ill-conditioning of the eigenvectors (becoming parallel) or of the eigenvalues (many degenerate near zero values) or both [13,15].

The influence of sequence type on the frequency of pathological matrices originated in part from absence of some sequence states and potentially also from the reduced divergence of protein-coding DNA sequences. The exp_TAYL _algorithm exhibited the most striking difference between exonic and intronic sequences. As demonstrated by our artificial dinucleotide example, where we set multiple states to being unobserved, these failures arose from multiple unobserved sequence exchanges. The exp_TAYL _algorithm should therefore not be used on data sets where this may arise. The exp_EIG _algorithm also exhibited a difference in failure rates between the two sequence classes – ~10% trinucleotide matrices on intronic data, ~5% dinucleotide matrices and ~30% codon matrices on primate exonic data. Combined with the difference in error rates of dinucleotide matrices for positions 1+2 and 2+3 on exonic data, these results suggest that the ultimate biological cause of these failures is the suppressing influence of natural selection on substitution rates. Of the three codon positions, positions 1+2 are subjected to greater scrutiny by natural selection than positions 2+3. The result is that many of the dinucleotides exhibit similarly reduced substitutions in a matrix such that some of the eigenvectors may be almost parallel, resulting in a near-singular matrix. The fact that there is little difference in the matrix norm statistics between dinucleotide 1+2 and 2+3, yet the maximum eigenvector matrix condition numbers exhibit a similar order of magnitude to that observed for the trinucleotide cases (Tables 1 and 3), is consistent with this hypothesis [16]. The absence of such errors from the primate intron dinucleotide matrices (Table 2) follows, because the putative absence of selective constraint on intronic sequence would allow greater differentiation between the dinucleotide substitutions and thus result in matrices that were not close to singular. This argument also applies to the increased frequency of errors affecting codon matrices compared with the trinucleotide counterparts (Table 3). These results indicate that combinations of divergence and natural selection, such as those considered here, exist to which the exp_EIG _algorithm is not well suited.

We considered an alternate, but less likely, explanation for the distinct frequency of pathological matrices between the protein coding and intronic sequence classes – that they represent an artefact of the concatenation of protein coding sequences. Concatenation of sequences from protein coding genes subjected to distinct evolutionary and mutagenic processes could generate (or obscure) pathological matrices. We evaluated this possibility for dinucleotide matrices on individual protein coding sequence alignments for just one of the species triples (*Anabaena variabilis, Anabaena sp. *PCC 7120 and *Thermosynechococcus elongatus*). The results were consistent with those reported above regarding the occurrence of errors for exp_EIG _(Table 4). In contrast to the results from the concatenated alignments, there was a substantial error rate from exp_TAYL_. Consistent with our assessment from trinucleotide matrices from protein coding sequences, the failures were predominantly caused by the absence of some states, a case that was increased due to the much shorter length of the individual alignments. These properties were robust to whether exp_PADÉ _or exp_EIG _were used in the constrained optimisation step (Table 4). That all algorithms exhibited a consistently lower median error rate on the $\hat{Q}$ estimated using exp_PADÉ _(~0.0017) compared with those estimated using exp_EIG _(~0.0026) further supports the robustness of exp_PADÉ _.

**Table 4:** Exponentiation of individual matrices from protein coding exons from a triad of microbial species.

**Fit^1^**	**Data**	**Norm^2^**	**Cond^2^**	**ϵ_TAYL_^2^**	**ϵ_EIG_^2^**	**ϵ_PADÉ_^2^**	$E_{TAYL}^{3}$	$E_{EIG}^{3}$	**E_PADÉ_^3^**	**N^4^**
exp_PADÉ_	Dinuc 1+2	(0.13, 3.7e+02)	(10, 1.3e+18)	(6.2e-06, 1.2e+137)	(6.2e-06, 86)	(6.2e-06, 0.96)	85	141	0	5580
	Dinuc 2+3	(0.35, 5.8e+02)	(15, 9.4e+16)	(0.0017, 5.2e+141)	(0.0017, inf)	(0.0017, 0.97)	89	140	0	5580
exp_EIG_	Dinuc 1+2	(0.13, 2.6e+02)	(15, inf)	(1.4e-05, 1.5e+70)	(1.4e-05, 0.96)	(1.4e-05, 0.96)	86	137	0	5580
	Dinuc 2+3	(0.36, 5.4e+02)	(17, 2e+17)	(0.0026, 1.6e+133)	(0.0025, 1)	(0.0026, 1)	99	56	0	5580

The species were *Anabaena variabilis*, *Anabaena sp*. PCC 7120 and *Thermosynechococcus elongatus*.

^1 ^Fit – the algorithm used for the constrained estimation of **Q**; ^2 ^– median and maximum values; ^3 ^– Number of **P **matrices for the indicated algorithm with an invalid probability; ^4 ^– total number of matrices; inf – an infinite difference, typically arising from an exponentiation error.

Matrices pathological to an algorithm can also occur during numerical optimisation. What defines the frequency of such matrices is unclear but they do arise during optimisation. For ~1% of the primate intron trinucleotide **P **matrices, the Taylor series to the second element (which we used as the initial estimate for optimisation) were pathological to exp_EIG_. Whether occurrence of such matrices during an optimisation would affect the resulting solution is unclear.

Although exp_EIG _lends itself to caching, the utility of a global **Q**, and thus the importance of caching, diminishes when considering non-reversible models. Non-stationarity is implicitly a part of non-reversible phylogenetic models; this, by definition necessitates non-global **Q**. As evidenced by the computational speed summaries in Table 5, if numerous exponentiations are required then exp_PADÉ _has a clear performance advantage in addition to its numerical robustness.

**Table 5:** Exponentiation algorithm compute times.

**Alphabet**	**Algorithm**	**Time**
Nucleotide	exp_EIG_	0.2
	exp_TAYL_	0.4
	exp_PADÉ_	0.2
Dinucleotide	exp_EIG_	0.8
	exp_TAYL_	0.6
	exp_PADÉ_	0.4
Trinucleotide	exp_EIG_	11.2
	exp_TAYL_	7.6
	exp_PADÉ_	3.7

Mean compute time (milliseconds) from 100 different matrices.

## Conclusion

We have determined that matrices pathological to the most commonly applied matrix exponentiation algorithms exist in nature. The robust behaviour of the Padé with scaling and squaring algorithm, combined with its performance advantage for larger sequence alphabets, establishes this as the algorithm most suited to non-reversible models.

## Methods

### Sampling Sequences from Microbes

Most methods for inferring rate matrices perform best when the diagonal of the probability matrix is strongly dominant, i.e. when there are relatively few changes. Incorrect choices of the outgroup are also problematic, because such choices reverse the directionality of the evolutionary process. We therefore chose species triples where the sister taxa are substantially more related to one another than they are to the outgroup. Specifically, we required sister taxa to be at least 98% identical in their 16 S rRNA genes, and required the outgroup to be 90–98% identical, thus ensuring that all but the fastest-evolving proteins would have dominant diagonals in their **P **matrices and that the outgroup would be more different than the remainder. For each of these species triples, for each protein in one of the two sister taxa, its best BLAST hit in each of the other two taxa was recovered (blastp e-value 10^-10^). The best hits were then aligned using MUSCLE with default parameters and the nucleotide sequences from KEGG threaded back through the protein alignment to generate a nucleotide alignment.

### Sampling Sequences from Primates

We sampled primate sequence alignments from the Ensembl release 46 multiple-sequence alignments of *Homo sapiens *(human), *Pan troglodytes *(chimpanzee) and *Macacca mulatta (*macaque) genomes. Genomic coordinates for human protein coding genes were determined and Ensembl's PECAN genomic multiple alignments corresponding to the human coordinates selected for the species-set. Only genes for which sequence alignments were available for all three lineages were retained. Exonic sequence, indels and the simple repeat sequences were masked. Alignments were split into trinucleotide columns and any column containing characters other than ACGT were excluded and of the resulting filtered alignments those ≥ 50000 base pairs long were kept. This procedure resulted in ~1100 alignments of primate intron sequences.

Orthologous protein coding sequence (CDS) for the sampled human genes was extracted and aligned using the PyCogent progressive HMM alignment algorithm, with a codon substitution model [17]. All aligned codon columns containing non-nucleotide characters were removed. Due to the computational demands of fitting trinucleotide models, we concatenated 10 aligned genes in an arbitrary order (determined by the sort order of the Ensembl identifier of the human gene) resulting in ~100 alignments of concatenated primate protein coding genes.

All alignments were in trinucleotide frame, meaning that (starting from the first nucleotide) each triplet of nucleotide columns contained only 'real' trinucleotides. Due to the elimination of masked sequences, spanning between trinucleotides could create sequence elements not present in the original genome sequence. To avoid sampling artificial dinucleotides, alignments were split into trinucleotide columns first and either all first and second, or second and third positions were selected and concatenated to construct dinucleotide alignments. Nucleotide and trinucleotide alignments were derived without any modification.

### Estimating Q

For each alignment for a species triad, we used the known outgroup lineage to determine the direction of substitutions between sequence states for each ingroup lineage. Alignments were split into columns of non-overlapping motifs of either nucleotide, dinucleotide or trinucleotides with the trinucleotide based sampling of sequences taken into account for the dinucleotide sampling (see above). Columns in which all three sequences were different were excluded since the ancestral state of the ingroups cannot be determined. If the ingroups were identical to each other, but different from the outgroup, the ancestral state is taken as the ingroup state. If one of the ingroup lineages was identical to the outgroup, their state was taken as the ancestral state. These assignments of ancestral state were used to count the number of times motif *i *changed to motif *j *for each ingroup lineage separately. This procedure generated a 'counts' matrix for each lineage, for each alignment which can be converted into a **P **matrix by dividing the elements of each row by the row sum. The resulting **P **matrices can be non-reversible.

Estimation of **Q **from **P **matrices was done using a constrained optimisation procedure. All off-diagonal elements of **Q **(*q*_*ij*, *i *≠ *j*_) are free parameters while the diagonal elements are constrained to be *q*_*ii *_= -∑_*j*, *j *≠ *i *_*q*_*ij *_to ensure the row sums of the instantaneous rate matrix are 0. Constraining the *q*_*ij*, *i *≠ *j *_≥ 0, we minimised ||$\hat{P}$ - exp(**Q**)|| where the matrix exponential was computed by Padé (unless stated otherwise). This function was minimised using the PyCogent [18] Powell optimiser with tolerance of 10^-6 ^and a maximum of 5 restarts. We used the Taylor series to the second element, computed as **P **minus the identity matrix, as the starting values for the numerical optimisation. The number of free parameters for **Q **were *N *× (*N *- 1) (*N *is the number of motifs in the alphabet): 12 for nucleotides; 240 for dinucleotides; and 4032 for trinucleotide matrices. Due to the very large number of free parameters in the trinucleotide models, and computational times required to estimate some **Q **of ~24 hours (for the microbial samples), not all alignments were used. Codon matrices were derived for the protein coding sequences by removing the unobserved states which corresponded to the trinucleotides encoding stop codons.

### Measuring matrix exponentiation error

In order to quantify the accuracy of the matrix exponentiation methods a number of measures are introduced. Firstly, there is the concept of a matrix norm. The matrix norm ||**Q**|| is a generalisation of the vector norm (length of a vector) and yields a measure of the effective size of a matrix **Q **in terms of the size of its elements [7]. We employed the Frobenius norm (often easier to compute than the induced norms) [7], which for the matrix **Q **is

(1) $$||Q||=\sqrt{\sum_{i=1}^{n}\sum_{j=1}^{n}\left|q_{ij}^{2}\right|}$$

where the *q*_*ij *_are the elements of **Q**. The distance between two matrices can therefore be computed as the norm of the difference between the matrices and equates to the square root of the sum of absolute squared differences. We define relative measures of exponentiation error as

(2) $$\begin{array}{l} \epsilon_{TAYL}=||\hat{P}-\exp_{\text{TAYL}}(\hat{Q})|| \end{array}$$

(3) $$\epsilon_{EIG}=||\hat{P}-\exp_{\text{EIG}}(\hat{Q})||$$

(4) $$\epsilon_{PADÉ}=||\hat{P}-\exp_{\text{PADÉ}}(\hat{Q})||$$

where $\hat{P}$ is the probability matrix estimated from the counts matrix and $\hat{Q}$ is the instantaneous rate matrix estimated from this $\hat{P}$ as described above.

In addition to the norm of the rate matrix, the performance of some of the matrix exponentiation methods may depend on the condition number cond_EV_(**Q**) of the eigenvector matrix of **Q **[6,7]. The eigenvector matrix condition number cond_EV_(**Q**) can be defined within the spectral matrix norm (2-norm) [19] as [7]

(5) $${\text{cond}}_{\text{EV}}(Q)=||U|{|}_{2}||U|{|}_{2}^{-1}=\sigma_{\max}(Q)/\sigma_{\min}(Q)$$

where **U **is the matrix of eigenvectors and *σ*_max_(**Q**) and *σ*_min_(**Q**) are the maximal and minimal singular values [7] of **Q **respectively.

### Software implementation

The PyCogent [18,20] built-in routines for determining counts matrices and matrix exponentiation routines were used, while the matrix statistics were computed using the python numerical library, Numpy version 1.0.4 [21]. All scripts used to undertake this study are available on request.

## Authors' contributions

HS contributed to implementation of the matrix error measurements and interpretation; VBY contributed to implementation of constrained optimisation method, study design and interpretation; SE contributed to study design; RDK contributed to study design, microbial genome data sampling and interpretation; GAH contributed to study design, implementation of constrained optimisation, data sampling and interpretation.

## Supplementary Material

Additional File 1 A pathological rate matrix example. An analytical example of a matrix pathological to eigendecomposition. Click here for file
