# Supplementary material for: Pathological rate matrices: from primates to pathogens
Source: BMC Bioinformatics. 2008 Dec 19;9:550. doi: 10.1186/1471-2105-9-550 (PMC2639438; doi:10.1186/1471-2105-9-550)
Supplement: Additional File 1 — A pathological rate matrix example. An analytical example of a matrix pathological to eigendecomposition. [file 1471-2105-9-550-S1.pdf]

Demonstration of a matrix pathological to $\exp_{\mathrm{EIG}}$

We illustrate the analytical conditions under which the eigendecomposition approach can fail for a 4x4 matrix. The following example is pathological to $\exp_{\mathrm{EIG}}$ because the eigenvector matrix is not invertible. We note that despite the clear pathological nature of this matrix, sample matrices with this form are typically computable because of numerical rounding errors, illustrating that matrices will be pathological to an algorithm as a consequence of both the analytical properties of the procedure and machine precision.

Let $\mu_1, \ldots, \mu_8$ be positive such that $\mu_1 + \mu_2 = \mu_5 + \mu_6 = \alpha$, $\mu_3 + \mu_4 = \mu_7 + \mu_8 = \beta$, but $\mu_1 \neq \mu_5$ and $\mu_3 \neq \mu_7$. Rate matrix

$$Q = \begin{bmatrix} - & \mu_2 & \mu_3 & \mu_4 \\ \mu_1 & - & \mu_3 & \mu_4 \\ \mu_5 & \mu_6 & - & \mu_8 \\ \mu_5 & \mu_6 & \mu_7 & - \end{bmatrix}$$

Let $\lambda = \alpha + \beta$. Solving the differential equation

$$\frac{dP(t)}{dt} = P(t)Q$$

yields an analytical expression for $P(t)$.

The equilibrium distribution is

$$\pi = \frac{1}{\lambda^2}(\mu_1\alpha + \mu_5\beta, \mu_2\alpha + \mu_6\beta, \mu_3\alpha + \mu_7\beta, \mu_4\alpha + \mu_8\beta)$$

Let

$$c = \begin{bmatrix} (\pi_3 + \pi_4)(\mu_6 - \mu_2) & \pi_4(\mu_7 - \mu_3) - \pi_3(\mu_8 - \mu_4) \\ \pi_1(\mu_2 - \mu_6) - \pi_2(\mu_1 - \mu_5) & (\pi_1 + \pi_2)(\mu_3 - \mu_7) \end{bmatrix}$$

Let each row of 4×4 A be π, and

$$B = \begin{bmatrix} c_{11} & -c_{11} & -c_{12} & c_{12} \\ c_{11} & -c_{11} & -c_{12} & c_{12} \\ c_{21} & -c_{21} & -c_{22} & c_{22} \\ c_{21} & -c_{21} & -c_{22} & c_{22} \end{bmatrix}$$

Then

$$P(t) = \mathrm{diag}(e^{-\lambda t}) + (1 - e^{-\lambda t})A + te^{-\lambda t}B$$

The eigenvalues of Q are 0 and $-\lambda$, with algebraic multiplicity 3. But

$$Q + \lambda\mathbf{I} = \begin{bmatrix} \mu_1 & \mu_2 & \mu_3 & \mu_4 \\ \mu_1 & \mu_2 & \mu_3 & \mu_4 \\ \mu_5 & \mu_6 & \mu_7 & \mu_8 \\ \mu_5 & \mu_6 & \mu_7 & \mu_8 \end{bmatrix}$$

is of rank 2, so Q is not diagonalisable over the complex numbers.
